# Vietnam National Survey on Parenteral Nutrition Practice in Preterm Neonates: Practice Status, Barriers, and Implications

**DOI:** 10.7759/cureus.61187

**Published:** 2024-05-27

**Authors:** Thu Tinh Nguyen, Phan Minh Nhat Nguyen, Thanh Thien Nguyen, Pham Minh Tri Nguyen, Duc Ninh Nguyen, Thi Hieu Vu

**Affiliations:** 1 Department of Pediatrics, University of Medicine and Pharmacy at Ho Chi Minh City, Ho Chi Minh City, VNM; 2 Department of Neonatology, Children's Hospital 2, Ho Chi Minh City, VNM; 3 Department of Neonatology, Ho Chi Minh University Medical Center, Ho Chi Minh City, VNM; 4 Department of Neonatology, Children’s Hospital 2, Ho Chi Minh City, VNM; 5 Comparative Pediatrics and Nutrition, Department of Veterinary and Animal Sciences, University of Copenhagen, Frederiksberg, DNK

**Keywords:** lipid, prematurity, parenteral nutrition, glucose, amino acids

## Abstract

Background: Due to high risks of feeding intolerance, preterm infants often receive parenteral nutrition (PN) to ensure sufficient nutrition and energy intake. However, there is a lack of data on the status of clinical PN practice and barriers among neonatal care units in low- to middle-income countries like Vietnam. This extensive survey explores the status and barriers of PN practice for preterm infants in neonatal units across Vietnam and identifies the practical implications of enhancing nutritional outcomes in preterm infants.

Methods: A multicenter nationwide web-based survey on PN practice in preterm infants was conducted across 114 neonatal units from 61 provinces in Vietnam.

Results: Among 114 neonatal units receiving a request for surveys, 104 units (91.2%) from 55 provinces participated. Neonatal units were categorized as level I (2/104, 1.9%), II (39/104, 37.5%), III (56/104, 53.8%), and IV (7/104, 6.8%). We found that the initiations of PN within the first hour and the first two hours of life occurred in 80.8% (84/104) and 95.2% (99/104) of the units, respectively. The early provision of amino acids, or AA (within the first day of life) and lipids (within two days of life) were documented by 85% (89/104) and 82% (84/104) of the respondents, respectively. The initial dose of AA ranged from 0.5 to 3 g/kg/day; the dose of AA less than 1 g/kg/day was reported by 7.7% (8/104) of the respondents; the maximum dose of AA ranged from 2 to over 4.5 g/kg/day, with 4 g/kg/day reported by 47.1% (49/104) of the respondents. The initial dose of lipids was between 0.5 and 2 g/kg/day, frequently 1 g/kg/day, reported by 51.9% (54/104) of the respondents; the target lipid dose ranged from 3 to 4 g/kg/day in 93.3% (97/104) respondents; the maximum target dose for lipid was 4 g/kg/day in 36.5% (38/104) of the respondents. The initial glucose dose was distributed as follows: 46.2% of respondents (48/104) administered 4 mg/kg/minute, 21.2% (22/104) used 5 mg/kg/minute, 28.8% (30/104) used 6 mg/kg/minute, and 3.8% (4/104) used 3 mg/kg/minute. Additionally, 48.1% of respondents (50/104) reported a maximum glucose infusion rate above 13 mg/kg/min and 19.2% (20/104) above 15 mg/kg/min. Nineteen percent (20/104) of the respondents reported a lack of micronutrients. Barriers to PN initiation included difficulty in establishing intravenous lines, the absence of standardized protocols, the lack of lipids and micronutrients, infections, and unavailable software supporting neonatologists in calculating nutrition paradigms.

Conclusion: This study's findings highlight the highly variable PN practice across neonatal units in Vietnam. Deviations from current practical guidelines can be explained by various barriers, most of which are modifiable. A monitoring network for nutritional practice status and a database to track the nutritional outcomes of preterm infants in Vietnam are needed.

## Introduction

Globally, approximately 4%-16% of total live births are preterm births (<37 weeks of gestation) [1], which leads to multiple serious complications with high mortality and morbidity during the neonatal period. These complications seem to be universal in both high-income and low-to-middle-income countries and contribute to around one-third of neonatal deaths [2]. Complications related to preterm birth also cause long-term health consequences, such as an increased risk of hypertension, insulin resistance, and neurodevelopmental disabilities later in life [3,4]. As the survival rate of preterm infants has increased in low- to middle-income countries like Vietnam, preterm birth likely imposes a greater burden and impact on society [4].

Recent clinical trials have shown that parenteral nutrition (PN) is important for clinical and neurodevelopmental outcomes in preterm infants. For instance, delayed or insufficient nutrient provision may lead to energy deficiency, elevating the risk of complications such as extrauterine growth retardation, cortical myelination deficiency, and neurodevelopmental impairment [3,5,6]. Ensuring timely and sufficient nutrition is key to enhancing survival rate and quality of life for premature infants [5,7].

Despite the availability of PN guidelines for preterm infants in high-income countries [8-10], it is unclear how clinical practice is implemented at neonatal care units in Vietnam. Reports from neonatal intensive care units (NICUs) worldwide have shown frequent inadequate nutritional intake in early life, and the reasons behind this are multifactorial [11-13]. These may include variable PN practices among units, but it is possible that preterm infants in low- to middle-income countries need a distinct PN guideline from the one applied in high-income countries [4].

Using this background, we conducted a survey to assess the status of and barriers to PN practice in Vietnamese NICUs. The findings aim to help develop solutions for enhancing PN practices for preterm infants in low- to middle-income countries like Vietnam.

This article was previously posted to the medRxiv preprint server on April 29, 2024.

## Materials and methods

A cross-sectional survey was designed to capture insights into the current practice status and barriers associated with PN among neonatologists. A survey questionnaire, developed using Microsoft Forms (Microsoft Corporation, Redmond, WA), included 29 multiple-choice questions and four optional free-text items, enabling comprehensive data gathering on the topic (see the Appendices). In addition, the responses were anonymous and did not include patient data. The study received ethical approval from the Institutional Review Board of the tertiary referral hospital, Children's Hospital 2 in Ho Chi Minh City (project no. 211/GCN-BVNĐ2).

Prior to the main survey, the questionnaire was pretested with pediatric postgraduate students to ensure clarity, detail, and a logical sequence of questions. This pilot survey provided valuable feedback that informed the refinement of the survey items. The list of target respondents included neonatal units from 61 provinces in Vietnam known to administer PN to preterm neonates. Two of the 63 provinces were excluded due to a lack of parenteral nutrition services. Pre-ordered PN is unavailable in Vietnam. Instead, PN is mixed either in the pharmacy or directly within units, depending on local policy and available facilities. Neonatologists adjust the orders for PN for each infant daily, typically during morning or afternoon rounds.

In total, 114 anonymous online questionnaires were distributed through email, messages, and social messaging platforms among NICUs across these 61 provinces over one month from June 1 to June 30, 2021. Responses were collected through Microsoft Forms. The initial communication for the survey detailed the elements of written consent, and the act of response was taken as consent to participate. This introductory message also advised that a representative from each unit, preferably a senior neonatologist, should complete the survey to avoid duplicate responses and more reliable data. Throughout the survey period, weekly reminders were sent by the data management team to maximize the response rate. The average time required to complete the survey, as pretested during the pilot with postgraduate students, was approximately five minutes.

All data were anonymized to protect the identities of the participating neonatologists. The classification of NICUs was based on the American Academy of Pediatrics (AAP) levels of neonatal care [14]. Level I (essential care) NICUs stabilize, when necessary, newborns <35 weeks and provide care for newborns born at ≥35 weeks of gestation. Level II (specialty care) include Level I, and also provide care for newborns born at ≥32 weeks of gestation and weighing ≥1500 g and provide mechanical ventilation for a brief duration (<24 h) or continuous positive airway pressure. Level III (intensive care) include Level II and also provide care for infants born at all gestational ages, and provide critical care (advanced respiratory-cardiovascular support: mechanical ventilation, surfactant, inhaled nitric oxide, or iNO) and surgical care. Level IV (regional NICU) include Level III, and also provide surgical repair in complex congenital or acquired conditions (e.g., complex congenital cardiac malformations) and provide outreach education.

For statistical analysis, data were first exported to Microsoft Excel and subsequently imported into R statistical software (RStudio 2023.12.1+402 for MacOS; R Foundation for Statistical Computing, Vienna, Austria). The data on barriers to PN were collected from free text responses, which had to be read through and coded into variations. Descriptive statistics were utilized to summarize the survey findings, and the qualitative data were presented in terms of both the frequency and percentage of responses, as well as the identification of barriers encountered in PN practice.

## Results

Representatives from 104 NICUs across 55 provinces in Vietnam answered the survey. The response rate was 104 responses out of 114 distributed surveys (91.2%). The participating NICUs were categorized based on levels of neonatal care: Level I, 2 out of 104 (1.9%); Level II, 39 out of 104 (37.5%); Level III, 56 out of 104 (53.8%), and Level IV, 7 out of 104 (6.8%) (see Table 1). The initiations of PN for preterm infants within the first hour of life and the first two hours of life occurred in 80.8% (84 out of 104) and 95.2% (99 out of 104) NICUs, respectively.

**Table 1:** Characteristics of neonatal intensive care units (NICUs) responding to the survey on parenteral nutrition (N = 104) Data are presented as N (%).

Characteristics		N (%)
Type of hospital	Government	88 (84.6)
	Private	16 (15.4)
Level of NICUs	Level I	2 (1.9)
	Level II	39 (37.5)
	Level III	56 (53.8)
	Level IV	7 (6.8)
Region	Ha Noi City	9 (8.7)
	Ho Chi Minh City (HCMC)	18 (17.3)
	North region (except Ha Noi)	16 (15.4)
	North central region	8 (7.7)
	Central region	8 (7.7)
	South region (except HCMC)	34 (32.7)
	South central region	11 (10.6)

Amino acids

Over 85% (89 out of 104) of the respondents provided parenteral amino acids (AA) within the first day of life, while 10% (11 out of 104) and 4% (4 out of 104) initiated AA on the second day and third day of life, respectively. The initial dose of AA ranged from 0.5 to 3 g/kg/day. The dose of AA less than 1 g/kg/day was reported by 7.7% (8 out of 104) of the respondents. The maximum dose of AA ranged from 2 to 4.5 g/kg/day, with 4 g/kg/day reported by 49 of 104 (47.1%) of the respondents (Table 2).

**Table 2:** Current practice of parenteral nutrition (amino acids and lipids) in neonatal intensive care units responding to the survey (N = 104) Data are presented as N (%).

		Number of units (%)	
Variables		Amino acids	Lipids
Initial time (hours), n (%)	Within 24	89 (85.6)	59 (56.7)
	24-48	11 (10.6)	26 (25.0)
	Above 48	04 (3.8)	19 (18.3)
Initial dose (g/kg/day), n (%)	0.5	08 (7.7)	36 (34.6)
	1.0	26 (25.0)	54 (51.9)
	1.5	35 (33.7)	07 (6.7)
	2.0	28 (26.9)	06 (5.8)
	2.5	2 (1.9)	-
	3.0	5 (4.8)	1 (1.0)
Maximum dose (g/kg/day)	2.0	1 (1.0)	3 (2.9)
	2.5	03 (2.9)	3 (2.9)
	3.0	08 (7.7)	37 (35.6)
	3.5	28 (26.9)	22 (21.2)
	4.0	49 (47.1)	38 (36.5)
	4.5	12 (11.5)	1 (1.0)
	>4.5	3 (2.9)	-

Lipids

A total of 82% (85 out of 104) of the respondents reported the initiation of parenteral lipids within two days of life; of those, 69.4% (59 out of 85) initiated it within the first 24 hours of life. The initial dose was between 0.5 and 2.0 g/kg/day, frequently 1 g/kg/day, reported by 51.9% (54 out of 104), except one respondent where the dose reported was 3 g/kg/day. One respondent (1%) reported that lipids were not available in the NICU. The target lipid dose of 3-4 g/kg/day was reported by 93.3% (97 out of 104) of respondents. Additionally, the maximum target dose for lipids was 4 g/kg/day for 36.5% (38 out of 104) of respondents (Table 2).

Glucose

The initial glucose dose was distributed as follows: 46.2% (48 out of 104) of the respondents used 4 mg/kg/minute, 21.2% (22 out of 104) used 5 mg/kg/minute, 28.8% (30 out of 104) used 6 mg/kg/minute, and 3.8% (4 out of 104) used 3 mg/kg/minute. Additionally, the maximum rates of glucose infusion above 13 mg/kg/min and 15 mg/kg/min were reported by 50 (48.1%) and 20 (19.2%) respondents, respectively. Nineteen percent (20 out of 104) of the respondents reported lacking parenteral micronutrients.

Barriers

**Figure 1:**
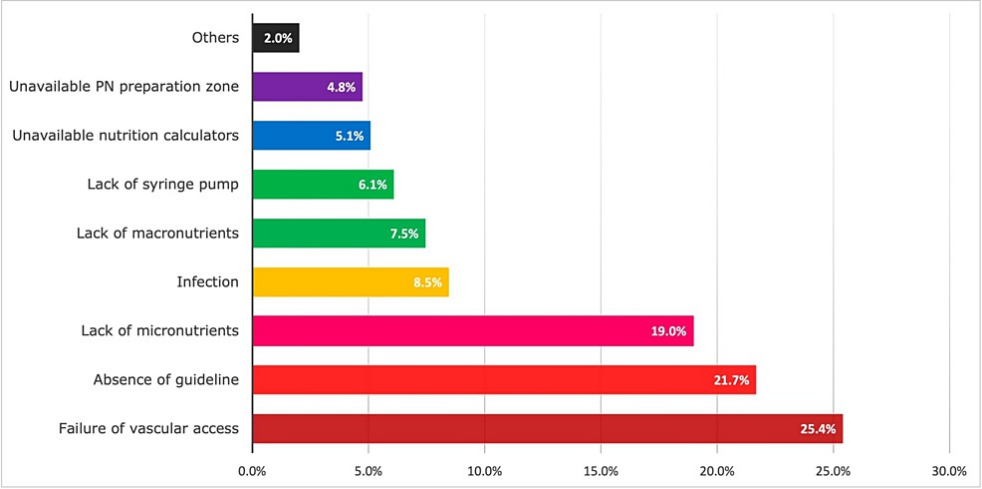
Main barriers affecting parenteral nutrition (PN) practice among neonatal intensive care units (NICUs) in Vietnam A total of 295 opinions received from 104 respondents were categorized into eight types of barriers. "Others" included early initiation of lipids and amino acids not allowed by the head of NICUs, a lack of staff responsible for PN, and severe comorbidities complicating PN orders. The percentages were calculated based on the total number of opinions (N = 295).

Figure 1 details the barriers that have deterred the respondents from applying their knowledge to current clinical practice. Among these, difficulties in establishing vascular access, the absence of local PN guidelines and lack of micronutrients are the most common reported barriers.

A total of 295 opinions received from 104 respondents were categorized into eight types of barriers. "Others" (2%) included early initiation of lipids and amino acids not allowed by the head of NICUs, a lack of staff responsible for PN, and severe comorbidities complicating PN orders (Figure 1).

## Discussion

It is well known that PN is associated with multiple clinical and neurodevelopmental outcomes in preterm infants [3,5,6,15]. The PN practices are highly variable among neonatal units, and units in low- to middle-income countries may need distinct PN guidelines [4]. Here, we conducted a national survey on PN practice across 55 provinces in Vietnam with 91.3% (95 out of 104) of NICUs from levels of care II to III, which are in the general hospitals. Many of these NICUs lack sufficient facilities and staff that care for preterm infants. The most recent European and US guidelines recommended that PN should be initiated early, within the first one to two hours of life in preterm infants [9,10,16,17], and our survey results reported a high level of compliance.

The survey documented that 85.6% (89 out of 104) of NICUs provided amino acids within 24 hours of life (on the PN was initiated), consistent with existing guidelines [9,10,16-18]. Still, the remaining NICUs (14.4%) deferred providing AA. Reasons for this include the need for medical consultation and concerns about kidney function of preterm infants. According to a study in premature infants born at <30 weeks of gestation, levels of creatinine remained in the normal range following PN with the recommended protein intake during the first three weeks of life [19]. In principle, the provision of AA aims to mimic the AA accretion by the fetus. The initial AA dose recommended in clinical trials and guidelines was 1-2 g/kg/day, which is expected to prevent a net negative nitrogen balance [9,18,20]. Our survey revealed that the initial dose of AA ranged from 1 to 2 g/kg/day in 85.6% (89 out of 104) NICUs. For the maximum dose of AA, this varies in previous clinical trials, from 2.5 to 4.5 g/kg/day [17]. Current evidence also shows limited difference in clinical outcomes between a maximum AA dose of 3.5 versus 4 g/kg/day [5,17]. Our survey showed that the maximum dose of AA was consistent with the recommendations (ranging from 3.5 to 4 g/kg/day) in 74% (77 out of 104) of the NICUs.

The provision of parenteral lipids aims to improve growth and prevent an essential fatty acid deficiency (EFAD). The survey documented the use of parenteral lipids in 99% (103 out of 104) of the NICUs. Lipids were initiated early on in the first two days of life as reported by 81.7% (85 out of 104) of the respondents. The remaining 18% (19 out of 104) of the NICUs started lipids on the third day of life because they believed that lipids need to be administered separately from PN. In fact, lipids can be infused along with parenteral nutrition via three-way stopcocks. The recommended initial dose of lipids ranges from 0.5 to 2 g/kg/day, whereas our study showed that 86.5% (90 out of 104) of the units used 0.5-1 g/kg/day. The maximal target dose of lipids was 4 g/kg/day for a minority of respondents, at 35.6% (37 out of 104). This minority could be explained by the low rate of extremely low birth weight infants in their NICUs. The specific composition of lipids might play a role of benefit in enhancing growth outcomes. The ideal lipid emulsion should contain n-6 and n-3 fatty acids (fish oil), antioxidant agents (e.g., tocopherol and monounsaturated fatty acids), and medium-chain fatty acids [9,16]. Clinically, most units use 20% lipid emulsions (instead of 10%) as they provide more energy than 10% emulsions (2 kcal/ml vs. 1.1 kcal/ml), and contain a lower phospholipid content, with a limited increase in plasma triglycerides [10].

The survey documented the maximal target dose of glucose over 13 mg/kg/min in 48.1% (50 out of 104) respondents. The glucose infusion of >12 mg/kg/min may overwhelm the maximal capacity of glucose oxidation of the liver in preterm infants. In contrast, the rate of 4 mg/kg/min is the estimated rate of glucose use by the brain [8]. In addition, a normal daily breastmilk intake of about 150 ml/kg/day can provide a glucose rate of 4 mg/kg/min, and this was the threshold for initial glucose infusion documented by 46.2% (48 out of 104) of NICUs. Despite the demand for high energy for growth in very low birth weight infants, they do not often tolerate the recommended glucose dose during the first few days of life. These infants have high risks of hyperglycemia, explaining the balance of using an initial glucose infusion of 4 mg/kg/min. On the other hand, other groups of preterm infants are recommended to start with a glucose regimen of 5-6 mg/kg/min [8].

There were multiple recorded barriers affecting PN practice among NICUs in Vietnam. Most of the mentioned barriers include failure of venous access, lack of parenteral protocols or consensus guidelines, unavailable methods of PN calculation, and unavailable micronutrient products. Some barriers can actually be resolved in the near future by developing national PN guidelines, improving peripherally inserted central catheter (PICC) insertion procedures, and developing computer software to support decision-making [21]. Simultaneously, there is an urgent need for the establishment of a collaborative nutritional network and a national database to evaluate nutritional outcomes in the future.

Our survey has some limitations too. Due to the limited number of NICU Level I and Level IV facilities included in the survey, the results may have restricted generalizability. The small sample size from these NICU levels may not fully represent the diverse practices and challenges experienced across all neonatal care units in the country. Consequently, the findings might not be applicable to all NICU settings, potentially limiting the broader applicability and impact of the survey's conclusions.

## Conclusions

The findings of this study highlight great variations in PN practice in Vietnam, particularly in amino acids and micronutrient provision. Deviations from current practical guidelines can be explained by various barriers, most of which are modifiable. There is an urgent need to establish a monitoring network for nutritional practice status and develop a database to track the nutritional outcomes of preterm infants in Vietnam. Also, surveys should be conducted to determine the effects of interventions on barriers and nutritional outcomes in preterm infants in Vietnam.

## Appendices

Questionnaire distributed to survey participants in neonatal intensive care units

General Information

1.    What is the name of the hospital where you work?

----------------------------------------------------

2.    Which level of care is provided in your neonatal department or neonatal intensive care unit (NICU)?

Level I     Level II     Level III     Level IV

[Notes:

Level I (Essential Care): stabilize and provide care for neonates ≥ 35 weeks of gestation. Stabilize neonates < 35 weeks. 

Level II (Specialty Care): Level I care + provides care for neonates ≥ 32 weeks of gestation and weighing ≥ 1500g. Provide mechanical ventilation for a brief duration (<24 hours) or continuous positive airway pressure.

Level III (intensive care): Level II care + provides care for neonates born at all gestational ages. Critical care (advanced respiratory- cardiovascular support: mechanical ventilation, surfactant, iNO) and surgical.

Level IV (Regional Neonatal Intensive Care Unit): Level III Care + Provide surgical repair of complex congenital or acquired conditions [(e.g., complex congenital cardiac malformations), provide outreach education]

3.    Is your neonatal department or neonatal intensive care unit at a government or private hospital?

Government               Private

4.    In which province is your neonatology department or NICU located?

----------(Free text) --------------

5.    Does your unit provide parenteral nutrition (PN) for neonates?

Yes             No (stop the survey).

Awareness of Parenteral Nutrition (PN)

In this section, please choose according to your awareness or opinion of PN in preterm neonates.

6.    When do you start PN for a preterm infant admitted to your unit or department?

In the first hour

In the first 2 hours

2-6 hours after birth

> 6-12 hours after birth

> 12 hours after birth

7.    Which ingredients do you use for PN? (More than one option may be chosen.)

Glucose

Calcium (Ca)

Sodium (Na)

Potassium (K)

Amino acids

Lipids

None

8.    In your opinion, when do you initiate amino acids for preterm neonates?

Within 24 hours after birth

24-48 hours after birth

Above 48 hours after birth

9.    What is the appropriate starting dose of amino acids (g/kg/day) for preterm neonates?

0.5     1     1.5     2     2.5     3

10.    What is the recommended maximum dose of amino acids (g/kg/day) for preterm neonates?

2     2.5     3     3.5     4     4.5     Above 4.5

11.    When should lipids be initiated for preterm neonates?

Within 24 hours after birth

24-48 hours after birth

Above 48 hours after birth

12.    What is the appropriate starting dose of lipids (g/kg/day) for preterm neonates?

0.5     1     1.5     2     2.5     3

13.    What is the recommended maximum dose of lipids (g/kg/day) for preterm neonates?

2     2.5     3     3.5     4     4.5     Above 4.5

14.    What is the appropriate starting dose of glucose (mg/kg/min) for preterm neonates?

3     4     5     6     Above 6

15.    What is the maximum rate of glucose infusion (mg/kg/min) for preterm neonates?

10     12     14     16     Not mentioned     Others (free text)

16.    In addition to the three main nutrients, which are glucose, protein, and lipids, please list, in order of priority, three substances that are considered essential for effective PN. 

1. ----- 2. ----- 3. ----- (free text) …….

Current Practice of Parenteral Nutrition (PN) 

In this section, please choose according to the actual practice in your department or unit.

17.    In your department or unit, right after birth, when is PN usually started for premature neonates?

Within the first hour

Within the first 2 hours

2-6 hours after birth

> 6-12 hours after birth

Above 12 hours after birth

18.    In the case of the above sentence, which ingredients of PN will be given? (More than one option may be chosen.)

Glucose

Calcium (Ca)

Sodium (Na)

Potassium (K)

Amino acids

Lipids

None 

19.    When are amino acids provided to premature neonates in your department or unit?

Within 24 hours after birth

24-48 hours after birth

After 48 hours after birth

20.    What is the starting dose of amino acids (g/kg/day) for premature neonates in your department or unit?

0.5     1     1.5     2     2.5     3

21.    What is the maximum dose of amino acids (g/kg/day) for premature neonates in your department or unit?

2     2.5     3     3.5     4     4.5     Above 4.5

22.    When are lipids provided to premature neonates (g/kg/day) in your department?

Within 24 hours after birth

24-48 hours after birth

Above 48 hours after birth

23.    What is the starting dose of lipids (g/kg/day) for premature neonates in your department or unit?

0.5     1     1.5     2     2.5     3

24.    What is the maximum dose of lipids (g/kg/day) for premature neonates in your department or unit?

2     2.5     3     3.5     4     4.5     Above 4.5

25.    What is the starting dose of glucose (mg/kg/min) for premature neonates in your department or unit?

3     4     5     6     Others (free text)

26.    What is the maximum rate of glucose infusion (mg/kg/min) for premature neonates in your department or unit?

10     12     14     16     Not mention     Others (free text)

Obstacles in Parenteral Nutrition

In this section, please choose your answer according to barriers/difficulties/obstacles in the practice of parenteral nutrition in your department or unit.

27.    Are there any hesitations about the early administration of amino acids as recommended for PN in preterm neonates?

No               Yes (detail the reasons).

28.    Are there any hesitations in providing the high dose of amino acids recommended for PN in preterm neonates?

No               Yes (detail the reasons).

29.    Are there any hesitations about the early administration of lipids as recommended for PN in preterm neonates?

No               Yes (detail the reasons).

30.    Are there any difficulties with central venous vascular access (including PICCs and CVCs)?

No               Yes (detail the reasons).

31.    Is there a lack of micronutrients or macronutrients for PN in preterm neonates?

No               Yes (listed these nutrients)

32.    In addition to the barriers, difficulties, and obstacles mentioned above, are there any other barriers, difficulties, or obstacles to the practice of parenteral nutrition in your department or unit? Please mention them.

------------------------------------------

33.    What are the top three barriers to parenteral nutrition at your workplace? Please detail your opinions:

------------------------------------------
